# An Evaluation of the Pedestrian Classification in a Multi-Domain Multi-Modality Setup

**DOI:** 10.3390/s150613851

**Published:** 2015-06-12

**Authors:** Alina Miron, Alexandrina Rogozan, Samia Ainouz, Abdelaziz Bensrhair, Alberto Broggi

**Affiliations:** 1 ISR Laboratory, University of Reading, Reading RG6 6AY, UK; 2 INSA Rouen/LITIS laboratory - EA4108, Saint-Etienne du Rouvray 76801, France; E-Mails: alexandrina.rogozan@insa-rouen.fr (A.R.); samia.ainouz@insa-rouen.fr (S.A.); abdelaziz.bensrhair@insa-rouen.fr (A.B.); 3 VisLab, University of Parma, Parco Area delle Scienze 181A, 43100 Parma, Italy; E-Mail: broggi@vislab.it

**Keywords:** infrared pedestrian classification, multi-domain, multi-modality, multi-cue, feature comparison, intensity self-similarity, stereovision, benchmark

## Abstract

The objective of this article is to study the problem of pedestrian classification across different light spectrum domains (visible and far-infrared (FIR)) and modalities (intensity, depth and motion). In recent years, there has been a number of approaches for classifying and detecting pedestrians in both FIR and visible images, but the methods are difficult to compare, because either the datasets are not publicly available or they do not offer a comparison between the two domains. Our two primary contributions are the following: (1) we propose a public dataset, named RIFIR , containing both FIR and visible images collected in an urban environment from a moving vehicle during daytime; and (2) we compare the state-of-the-art features in a multi-modality setup: intensity, depth and flow, in far-infrared over visible domains. The experiments show that features families, intensity self-similarity (ISS), local binary patterns (LBP), local gradient patterns (LGP) and histogram of oriented gradients (HOG), computed from FIR and visible domains are highly complementary, but their relative performance varies across different modalities. In our experiments, the FIR domain has proven superior to the visible one for the task of pedestrian classification, but the overall best results are obtained by a multi-domain multi-modality multi-feature fusion.

## 1. Introduction

The main purpose of constructing intelligent vehicles is to increase the safety for all traffic participants. In this context, pedestrian protection systems have become a necessity. However, passive components, like airbags, are not sufficient: active safety technology assisting the driver in the prevention of a crash is vital. Therefore, a system for pedestrian detection plays a fundamental role. Motivated by the importance of such systems, there exists an extensive amount of work done in connection with this field in the last fifty years, but an efficient, robust and real-time solution does not exist yet.

Depending on the sensors used to acquire the road scene and the environment information, there exist different systems for pedestrian detection. Although active sensors, like Light Detection And Ranging (LiDAR) [1–3], work independently of ambient light and give as output a 3D map, they tend to have a low resolution that makes the task of pedestrian hypothesis classification difficult, which is an important module of a pedestrian detection system. Active systems are currently quite expensive, although the processing algorithms are basic. Passive sensors, represented by visible and infrared cameras, due to the low cost and high resolution, are by far the most used sensors for pedestrian detection systems, but they require complex processing algorithms.

A pedestrian detection involves the following steps: hypothesis generation (for mono-cameras in the visible domain, this is usually performed by sliding window techniques; in mono-cameras with thermal information using far-infrared wavelengths, because pedestrians will usually appear as bright regions, the hypotheses are generated using threshold-based methods; in stereovision, the hypotheses are generated starting from the disparity map); hypothesis verification, either by heuristics ([4,5]) or, most often, by classification techniques, allowing one to remove false positive hypotheses. Some pedestrian detection systems have a third step of tracking, for example using Kalman particle filters, in order to improve the hypotheses generation step or to assist the final decision making module based on pedestrian trajectory estimation [6].

A pedestrian detection system can be evaluated using either a per-image measure (this is called detection), as in [7], or a per-window measure, which is usually referred to as classification, preferred in [8]. Each technique has its advantages, but in what follows, for this article, we have chosen the classification context, because it is necessary first to analyse the relative performance of features and modalities in a multi-domain setup, independently of the pedestrian hypothesis generation step.

The objective of this article is to study the problem of pedestrian classification across different light spectrum domains (visible and far-infrared (FIR)) and modalities (intensity, depth and motion).

This article is organized as follows. In Section 2 is presented related work in visible and far-infrared domains in a multi-modality context. Section 3 overviews our main contributions. In Section 4, we present existing datasets for pedestrian detection and classification and also introduce a new dataset that we have acquired and annotated using both a visible and an FIR camera, which we call RIFIR. In Section 5, we evaluate different features over two domains and three modalities, followed by a discussion in Section 6. We conclude in Section 7.

## 2. Related Work

There is a significant amount of research work in the domain of pedestrian detection and/or classification. In [9], a review of pedestrian safety and collision avoidance systems is presented, which includes infrastructure enhancements. Pedestrian detection approaches are classified according to type and sensor configurations. In [10] is presented another survey that treats the task of pedestrian detection for advanced driver assistance systems (ADAS), but only describing the components of different pedestrian detection systems without a performance benchmark on a unique dataset.

A few surveys make a direct comparison of different systems at the feature and classifier levels on visible images. For instance, in [11], the authors describe the components of a pedestrian detection system, and a few representative systems are compared on the same dataset. Thus, the Daimler Pedestrian Detection Benchmark combines the wavelet features with the AdaBoost classifier, the histogram of oriented gradients (HOG) with a Support Vector Machine (SVM) classifier and the shape-texture model with neural networks using local receptive fields. They conclude that the HOG/SVM approach outperforms all of the other considered approaches. In [7] is proposed a challenging monocular visible dataset (Caltech database), along with an extensive comparison of different pedestrian detectors. It was shown that all of the top algorithms use in one way or another motion information.

Recently, a new direction of research for pedestrian classification and detection is represented by the combination of different features and modalities, extracted from the visible domain, such as intensity, motion information from optical flow [12] and depth information given by the disparity map [13].

### 2.1. Multi-Modality in the Visible Domain

Most of the existing research works use depth and motion for hypothesis generation, by constructing a model of the road scene geometry. For example, in [14], stereovision is used in order to segment the image into regions of interest, followed by a classification step based on geometric features computed from a 3D point cloud. In [12], motion information is used to extract regions of interest in the image, followed by shape-based detection and texture-based classification. In [15], stereo depth cues, ground-plane estimation and appearance-based object detection are integrated. Stereo-based region of interest (ROI) generation is also used in [16], along with shape-based detection, texture-based classification and (dense) stereo-based verification. In [13], a method for object detection and pedestrian hypothesis generation based on 3D information is proposed, and a motion-validation method to eliminate false positives among walking pedestrian hypothesis is used.

Rather than just using depth and motion as cues for hypothesis generation, a few research works began integrating features extracted from these modalities directly into the classification algorithm. For instance, in [17], the use of a histogram of oriented flow (HOF) is proposed in combination with the well-known HOG for human classification. In [18], a high level fusion of depth and intensity is proposed utilizing not only the depth information for the hypotheses generation step, but also extracting discriminative spatial features (gradient orientation histograms and local receptive fields) directly from (dense) depth and intensity images for classification purposes. Both modalities are represented in terms of individual feature spaces. In [19], motion estimation is incorporated, using HOG, Haar and HOF. A combination of HOF and HOG, along with other intensity-based features, with promising results on a challenging monocular dataset, Caltech [7], is described in [20]. In [21], the combination of HOG, HOF and a HOG-like descriptor applied on the disparity field that they call HOS, along with a proposed disparity statistic (DispStat) features is proposed. Most of these articles have used just one feature family applied on different modalities, and they lack an analysis of the relative performance of different feature families computed across different modalities.

The first extensive analysis was made in [22], where the authors proposed a new dataset for pedestrian classification and different modalities are combined, e.g., intensity, shape, depth and motion, extracting HOG, local binary patterns (LBP) and Chamfer-distance features. Moreover, a mixture-of-expert classification framework is proposed in [8] in order to integrate all of these features and to cope with large data.

### 2.2. Multi-Modality in the FIR Domain

In addition to multi-modality fusion (intensity, depth, motion) in the visible domain, several studies use stereovision in the far-infrared domain. For example, in [23], a four-camera system is used (two visible cameras and two far-infrared) and two dense disparity maps are computed: one in the visible and one in far-infrared domains. They use the information from the two disparity maps through the computation of v-disparity [24] in order to detect obstacles and generate pedestrian hypotheses. This work is extended in [25], where HOG-like features are computed on visible, infrared and a disparity map and then fused. Unfortunately, the tests performed by [23,25] were on a relatively small dataset, with a maximum number of five pedestrians in an image, where no other obstacles beside the pedestrians were present.

In [26,27], the authors proposed a system for pedestrian detection in stereo infrared images based on warm area detection, edge-based detection and v-disparity computation. Stereo information is used just to refine the generated hypothesis and to compute the distance and size of detected objects, but it is not used in the classification process.

Overall, there exists an important amount of work around multi-modality in the visible domain, and only a few articles that treat this problem in the FIR one; however, to our knowledge, there does not exist yet a study that integrates both multi-domain and multi-modality aspects.

## 3. Contributions

In this article, our objective is to analyse the relative performance of the state-of-the-art features, including a feature representation the we proposed in our previous work [28], called intensity self-similarity, for pedestrian classification in a multi-modality multi-domain setup. Our main contribution is that we considered the intensity, depth and flow modalities, on the multi-domain axis, considering in addition to the visible domain, the far-infrared one. Moreover, as a benchmark, we propose a public dataset, RIFIR, that contains annotated pedestrians on both FIR and visible images. We have also carried out extensive experiments on other several databases. We start by evaluating our system performance in the visible domain using the well-known Daimler Multi-Cue dataset. This has allowed us to reference the multi-modal features we used in comparison with the state-of-the-art ones on visible images. We also test the considered features on a public FIR dataset, OlmedaFIR-Classif. We then compare the relative multi-domain feature performance on two datasets, containing, for the given road scene, both visible and FIR images, the proposed RIFIR and the Parma-Tetravision. In the end, we test different fusion strategies between multi-modality features computed across visible and FIR domains.

Our intention is to provide an extensive benchmark framework, which should allow us to answer the following questions:

- (Q1) Is the FIR domain better than the visible one for the pedestrian classification in the daytime?
- (Q2) Do the state-of-the-art feature families in the visible domain have the same relative performance when tested in FIR?
- (Q3) Is the intensity self-similarity (ISS) feature family appropriate for pedestrian classification in the considered modalities (intensity, depth, flow) in the visible *versus* FIR domains?
- (Q4) What is the relative gain of multi-domain/multi-modality fusion?

## 4. Datasets

There exist several datasets that are publicly available and commonly used for pedestrian classification and detection in the visible domain. In what follows, we briefly present a few widely-used datasets. A more detailed comparison is made in [7]. INRIA [29] is a well-established dataset, but has a relatively small number of images. the NICTA dataset [30] consists mostly of images taken with a digital camera having cropped bounding boxes (BBs) as training and testing sets. Other datasets like, Caltech [7], Daimler Monocular [11], Daimler Multi-Cue [22], ETH [15] and KITTI [11], are all captured in an urban scenario with a camera mounted on a vehicle or on a stroller (as in the case of ETH).

Caltech [7] is one of the most challenging monocular databases, having a huge number of annotated pedestrians for both training and testing datasets. Daimler Monocular [11] provides cropped BB of pedestrians in the training set and road sequences of images for the testing. ETH [15] is a dataset acquired mostly on a side walk using a stroller and a stereovision setup; thus, it has both temporal information (images are provided in a sequence) and the possibility of using the disparity information. The KITTI object dataset [31] is a newer dataset that contains stereo images with annotated pedestrians, cyclists and cars. Although it does not allow using temporal information, there is the possibility of using 3D laser data. An interesting multi-modal dataset is Daimler Multi-Cue [22]. It contains cropped pedestrian and non-pedestrian BB, but with information from the visible domain and depth and motion modalities.

Although there exists a reasonable number of challenging public benchmark datasets for pedestrian detection in the visible domain, in the case of FIR images, most of the datasets are not publicly available. In Table 1, we present an overview of existing pedestrian datasets, along with the RIFIR database we propose. Datasets like that proposed by [32–35] focus mostly on surveillance applications; therefore, they use a fixed-camera setup. These kinds of datasets do not present the same challenges as images taken with a mounted camera in a vehicle for ADAS applications, because techniques like background subtraction could be applied, which can simplify the problem of pedestrian detection. Consequently, we do not use them in our experiments.

Recently in [36], was proposed a dataset acquired with an Indigo Omega camera, having an image resolution of 164 × 129. (We will further refer to this dataset as OlmedaFIR-Classif.) The dataset is divided into two parts: one that addresses the problem of pedestrian classification (OlmedaFIR-Classif) and the other one the problem of pedestrian detection (OlmedaFIR-Detection). Unfortunately, it does not contain any information from the visible spectrum, therefore making a complete assessment of system performance in FIR versus visible domain impossible. We could therefore only use it as a feature family benchmark in the FIR domain.

An interesting dataset that contains both FIR and visible images is proposed by [37]. Unfortunately, the dataset has just a small number of annotations (around 1000 pedestrian BB); therefore, it might not provide statistically-relevant results, and it is not publicly available (this dataset is maintained by VisLab and terms and conditions for usage may apply http://vislab.it/). Firstly, we have extended the annotations on the dataset proposed by [37]. We will further refer to the extended dataset as Parma-Tetravision. Secondly, we propose a new public dataset for pedestrian detection and classification in FIR images. It consists of sequences acquired in an urban environment with two cameras (FIR and colour) mounted on the top of a vehicle. We will further refer to the proposed dataset as RIFIR (the dataset is publicly available at the web address www.vision.roboslang.org). Moreover, for the two datasets, we have identified and labelled the occluded pedestrians (occluded area <50%; see Table 1).

In what follows, we will present the characteristics and statistics for the datasets we used in our experiments: Parma-Tetravision and RIFIR.

### 4.1. Parma-Tetravision Dataset

The Parma-Tetravision dataset contains information taken from two visible and two infrared cameras and was provided to us by VisLAB laboratory [37]. In a previous work [38], around 1000 pedestrians BBs were annotated, which is not large enough for a fair comparison of pedestrian classification system performances. Thus, we have extended the annotation to include a much larger number of images and manually annotated BB for both training and testing (for training, we have used Sequences 1 and 5 from the dataset; while for testing, Sequences 2 and 6.).

For the final dataset used for the problem of pedestrian classification, we have retained only those BBs that have a height above 32 *px*, are visible in both cameras and do not present major occlusions, where the occluded area is larger than 50%. Therefore, in the end, we have 6264 pedestrian BBs for training and 5743 pedestrian BBs for testing. These examples do not contain BBs generated using techniques, like mirroring. See Table 2 for an overview of the dataset.

**Figure 1:**
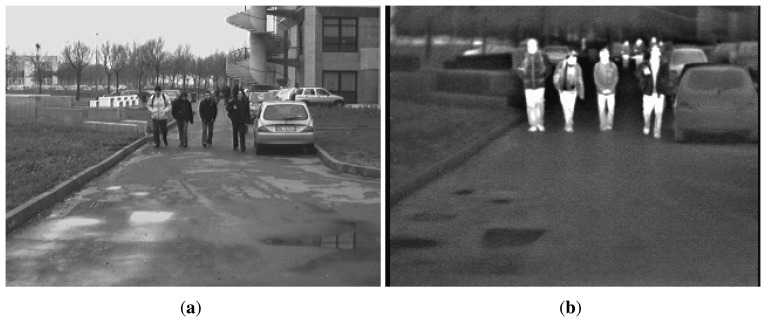
Images examples from the Parma-Tetravision dataset: (**a**) visible spectrum; (**b**) far-infrared spectrum.

In Figure 1 is presented an example of images from the Parma-Tetravision dataset.

It has to be mentioned that the resolution of the stereovision cameras was of 640 × 480 pixels, but the visible images have then been resized to 320 × 240 pixels for computation speed up purposes. It becomes thus identical to the resolution of the FIR cameras. Moreover, the field of view (FOV) of the stereovision cameras was of 0.44 × 0.34 radians, although the FOV of the FIR cameras was 0.30 × 0.24 radians. The scene registered with the visible camera is thus larger compared with the one registered with the FIR camera, as Figure 1 shows, and the obstacles do not have the same size in both types of images: the obstacles from the IR images are smaller than those from the visible images. Moreover, because of the larger FOV of the visible camera, obstacles appearing on the very left or right side and at the top part in the visible image may not appear in the IR image (they are out of the FOV of the FIR camera), and they may not have a correspondent in the FIR image. Such a situation could be handled by a pedestrian detection system with an adapted visible-FIR fusion scheme, by using a null weight for the FIR spectrum [39], when the classification decision will be taken only based on the visible information. Still, as could be seen in the following images, these obstacles correspond to those that are not in danger, because they are generally out of the vehicle trajectory.

The Parma Tetravisionsystem has been the subject of a strong calibration process, which is essential to obtaining pertinent information about the registered scene. When the system is moving on the road, especially when the road trajectory is uneven or after long distances are reached, it is possible that the system may suffer a miss-calibration. When this issue becomes significant, it could be solved by a manual calibration of the system. Nevertheless, the multi-domain fusion scheme we propose in this paper, at the multi-modal feature level, is less susceptible to the non-calibrations of on-board cameras compared to the ones performed at the lowest level of information (*i.e.*, at the data or pixel level).

### 4.2. RIFIR Dataset

We have acquired the proposed RIFIR dataset with two cameras: one visible domain camera (colour) with a resolution of 720 × 480 and an FIR camera with a resolution of 640 × 480. We have acquired two sequences in two different image acquisition sessions in similar weather conditions; thus, one of them was used in training and the other one in testing.

**Figure 2:**
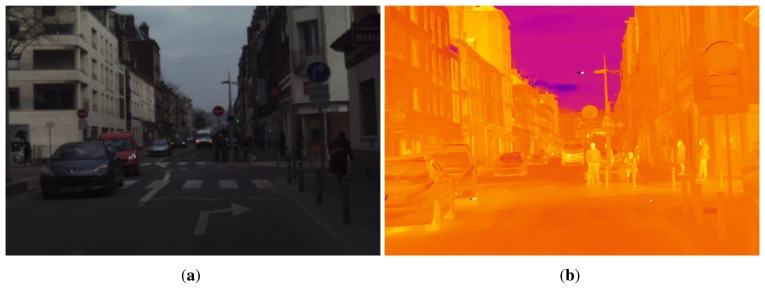
Image examples from the RIFIR dataset: (**a**) visible spectrum; (**b**) far-infrared spectrum.

As for the Parma Tetravision dataset, because of differences in camera optics and position, we have had to annotate the pedestrian independently in the visible and FIR images. In Figure 2, a pair of visible-FIR images from the RIFIR dataset is presented. The visible images in the RIFIR dataset are affected by noise because of a low level of light on a cloudy day, which is not the case for the visible images in the Parma Tetravision dataset.

Following the same methodology as in the case of the Parma-Tetravision dataset, for the constructed classification dataset, we have only considered those pedestrians with a height above 32 pixels, which are visible in both cameras, and do not present major occlusions. We have chosen this threshold height due to the fact that a pedestrian with a height of 32 pixels in infrared images is much smaller (with approximately 10 pixels) in the visible spectrum because of the difference in camera optics. In [7], the reasonable evaluation setting is set with all of the pedestrians with a height above 50 pixels. Because pedestrians in the infrared spectrum are easier to detect, we decided to lower this threshold limit to 32 pixels. As a consequence, there are 9202 pedestrian BBs for training and 2034 for testing. Moreover, since the pedestrian BBs in training and testing belong to disjoint acquisition sessions, one could never find the same unitary pedestrian in training and testing sets. This is much closer to reality than classification systems where all objects are melted together and then divided randomly between the training and testing sets. In what concerns the negative BBs, we have considered 25,608 in the training set and 24,444 in testing. Approximately 50% of these represent random BBs extracted from images. For the other half, we have applied a simple intensity threshold over the infrared images in order to obtain hot areas that might be confused with pedestrians. All of the negative BBs were generated on IR images and then projected into the corresponding visible images. See Table 3 for an overview of the dataset.

**Figure 3:**
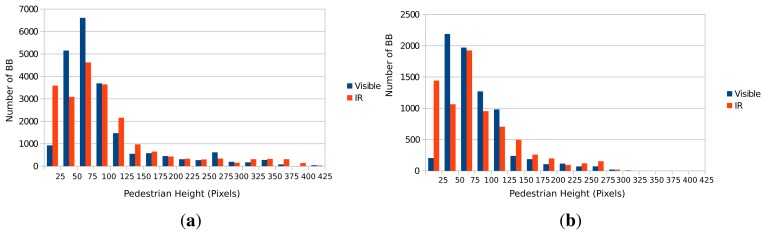
Pedestrian height distribution of training (**a**) and testing sets (**b**) for RIFIR.

**Figure 4:**
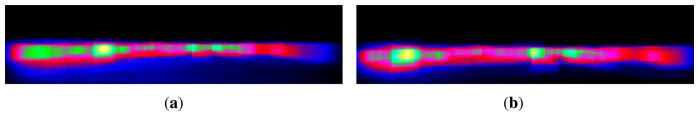
Heat map of training for the RIFIR dataset: (**a**) visible; (**b**) FIR.

In Figure 3 is presented the height histogram for the annotated pedestrian in both training and testing for the RIFIR dataset. While in the Parma-Tetravision dataset, most of the pedestrians had a height below 150 pixels, in the case of the RIFIR dataset, most of the pedestrians have a height below 100 pixels, thus making the dataset more challenging. The height histogram of annotated pedestrians is different in the two datasets, which is mostly due to differences in the road scenes. Furthermore, due to different camera optics, the visible camera has a wider FOV than the FIR camera, so objects that appear in one camera might not be visible in the other one. Moreover, as shown in Figure 4 , pedestrians tend to concentrate in the same region of the image, information that might be used to help and speed up the pedestrian detection algorithms [7].

## 5. Experiments

### 5.1. Preliminaries

In our experiments, we considered, for the visible domain, the state-of-the-art features: HOG [29], LBP [40] and LGP [41]. The motivation for using both HOG and LBP is that they are particularly robust features and have high complementarity Nevertheless, the LBP features are sensitive to local intensity variations and, therefore, could lead to many different patterns in a small region. This is a problem that may affect the performance of some classifiers. To overcome this problem, we also considered the LGP features, which generate constant patterns irrespective of local intensity variations along edges [41]. We added to the previous state-of-the-art characteristics a vector of intensity self-similarity (ISS) features. In our previous work [28], ISS features provided promising results on FIR images and are also complementary to HOG ones. The following experiments will try to answer if the state-of-the-art feature families (HOG, LBP, LGP and ISS) in the visible domain have the same relative performance when tested in the FIR domain, and *vice versa*.

For all considered databases (Daimler Multi-Cue, OlmedaFIR-Classif, Parma-Tetravision and RIFIR), in order to be consistent in the classification process, we have resized the annotated BBs to a size of 48 pixels in width and 96 pixels in height:

- HOG features are computed on cells of 8 × 8 pixels, accumulated on overlapping 16 × 16 pixel blocks, with a spatial shift of 8 pixels. This results in 1980 features.
- LBP and LGP features are computed using cells of 8 × 8 pixels, and a maximum number of 0 – 1 transitions of 2. This results in a number of 4248 features.
- ISS features are computed on cells of 8 × 8 pixels, a histogram size of 16 pixels and histogram difference. This results in 5944 features.

These features are fed to a linear SVM classifier. For this, we have used the library LIBLINEAR [42].

All of the results in this section are reported in terms of the ROC curve (false positive rate *vs.* detection rate), considering as a reference point the false positive rate obtained for a classification rate of 90%.

We have carried out extensive experiments with these features on the three considered databases. We start by evaluating each feature family individually on each modality (intensity, depth, motion) in the visible domain using the Daimler Multi-Cue dataset. Next, we have computed the relative performance between the considered features (HOG, LBP, LGP and ISS) on the visible domain from the Parma-Tetravision and RIFIR datasets. The same methodology is followed in the FIR domain, with the difference being that the state-of-the-art features are evaluated individually on the OlmedaFIR-Classif dataset. Then, the relative performance of these features is considered again on both multi-domain databases. Moreover, multi-modality (intensity, depth, motion) over multi-domain (visible, FIR) feature comparison and their fusion are performed on the Parma-Tetravision dataset. If on the Daimler Multi-Cue dataset, the disparity map (depth information) is already computed, for the Parma Tetravision dataset, we have used the algorithm proposed in [43] for efficient computation of the disparity map. The idea of this stereovision algorithm is to compute a prior on the disparities by forming a triangulation on a set of support points, thus reducing matching ambiguities. The main advantage of this algorithm is the computation speed, and it remains robust in poor-textured regions.

### 5.2. Feature Performance Comparison in the Visible Domain

We started by analysing the performance of the considered features in the visible domain on the Daimler Multi-Cue dataset.

**Figure 5:**
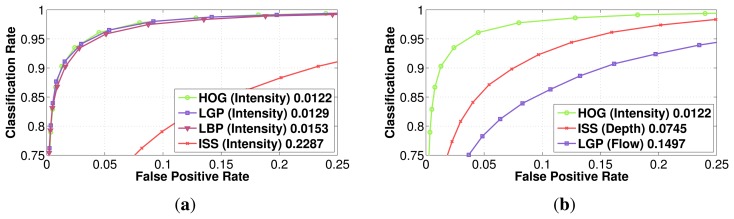
Daimler Multi-Cue: Individual classification performance comparison of different features on: (**a**) the intensity; (**b**) the best feature for each modality. The reference point is considered the false positive rate obtained for a classification rate of 90%.

In Figure 5, the performance of those different features is presented for the intensity modality, along with the best performing feature family on each modality (for Daimler Multi-Cue, a more in-depth feature comparison, along with fusion strategies, can be found at vision.roboslang.org).

In [8], the authors have also compared HOG and LBP features independently on each modality (intensity, depth and flow) on the visible spectrum and have drawn the conclusion that classifiers in the intensity modality have the best performance independent of the used features, by a large margin. Our experiment results show that most of the features computed on the intensity modality give indeed the best overall performance (HOG, LBP and LGP), but the ISS features perform better for the depth modality, while the LGP features outperform all of the previous features for the flow modality. On the whole, the best performance is obtained by the HOG features for the intensity modality, but followed very closely by LGP features computed also for intensity. In the depth modality, the proposed ISS features attain the lowest error rate, followed closely by the LGP features. Moreover, the HOG features, even if for the intensity, gave the best results; in the depth modality, they prove to be less robust than the ISS features or the texture-based features, like LGP and LBP.

**Figure 6:**
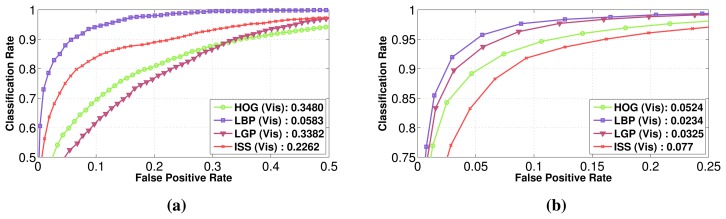
Feature performance comparison in the visible domain for the intensity modality on the datasets: (**a**) RIFIR; (**b**) Parma-Tetravision. The reference point is considered the false positive rate obtained for a classification rate of 90%.

It is to be noted that our HOG and LBP feature implementation reaches the same performance as the results presented in [8]. We decided as the next step to evaluate the considered features (HOG, LBP, LGP and ISS) in the visible domain on the datasets RIFIR and Parma-Tetravision. The results are reported in Figure 6. Throughout the article, the term visible in the figures refers to the intensity modality from the visible domain, while IR refers to the intensity modality from the FIR domain. LBP continues to be one of the most robust features for the intensity modality, obtaining a lower false positive rate for both the RIFIR and Parma-Tetravision datasets.

Thus, while ISS features manage to be more robust for the RIFIR dataset (in the context of noise), HOG and LGP perform better for the Parma-Tetravision dataset (higher quality images). In fact, RIFIR colour images have more noise than the grayscale images from Parma-Tetravision. This has a direct impact on the performance of features based on the gradient: HOG and LGP.

This first set of experiments on the visible domain allows us to emphasis the pertinence of ISS features, especially for the depth modality, to demonstrate that the considered features are complementary and that their relative performances are correlated with the characteristics of the images for a given image dataset.

### 5.3. Feature Performance Comparison for the FIR Domain

**Figure 7:**
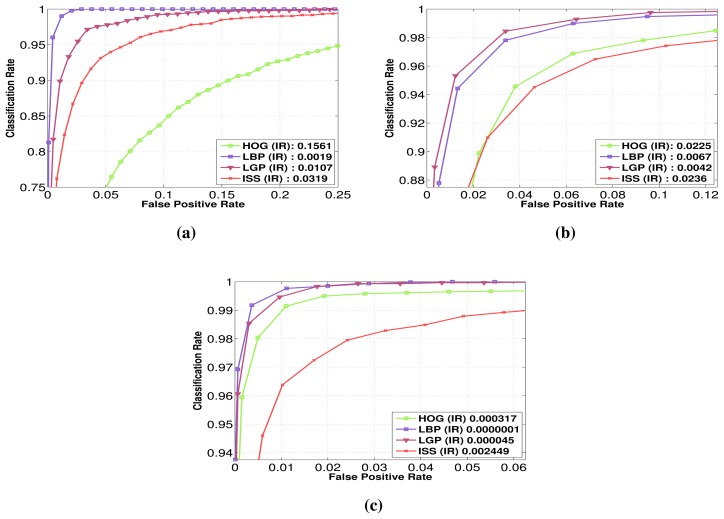
Performance comparison for HOG, local binary patterns (LBP), local gradient patterns (LGP) and intensity self-similarity (ISS) features in the FIR spectrum on the datasets: (**a**) RIFIR; (**b**) Parma-Tetravision; (**c**) OlmedaFIR-Classif. The reference point is considered the obtained false positive rate for a detection rate of 90%.

Having established the relative performance of the considered features in the visible domain for the intensity modality, we evaluate next their performance for the intensity modality, this time in the FIR domain. In Figure 7 are presented the performances of each feature family on the RIFIR dataset (Figure 7a), Parma-Tetravision (Figure 7b) and OlmedaFIR-Classif (Figure 7c).

For the RIFIR and OlmedaFIR-Classif datasets, the best performing feature is LBP, followed closely by LGP. For the Parma-Tetravision dataset, the best performing feature is LGP, followed closely by LBP. For the Parma-Tetravision and OlmedaFIR-Classif datasets, HOG features perform better than ISS, while on the RIFIR dataset, the situation is reversed.

In our opinion, the difference in performance between features comes from the fact that even if all three datasets were obtain using FIR cameras, there is a difference in sensors, road scenes and some environmental conditions, like outside temperature. It seems that the LBP and LGP features are more adapted than HOG and ISS features for the task of pedestrian classification in FIR images, taking advantage of local changes in intensity values. Nevertheless, because we consider that those are complementary, we will test this hypothesis with a fusion of features in Section 5.5.

The first set of experiments performed on the visible domain has shown that the relative performance between the feature families (HOG, LBP, LGP and ISS) varies from one modality to another among the considered modalities (intensity, depth, flow). This second set of experiments done in the FIR domain allows us to answer Q2, since the results have shown clearly that the feature families do not have the same relative performances in the visible over the FIR domain. In the following section, we will attempt to answer Q1, if the FIR domain is better than the visible one for pedestrian classification.

### 5.4. Visible vs. FIR

Having the performance of different features on both the Figure 6 and FIR domains (Figures 7a,b), we can now compare the two spectra. On both datasets, RIFIR and Parma-Tetravision, features computed on the FIR images have better performance than those computed on the visible domain, whichever the modality used (intensity, depth, flow). We withhold from drawing a definitive conclusion that for FIR images, the pedestrian classification will always perform better than for visible ones, because this could depend on the quality of the camera sensors used and also the optics.

The performance difference for the RIFIR dataset between FIR and visible is quite large for LGP and LBP with a factor of approximatively 30. HOG and ISS features computed on the intensity from the FIR domain result in a smaller number of false positives than the intensity from visible domains, with a factor of two, on both datasets.

### 5.5. Visible and FIR Fusion

In Section 5.4, we have shown that on the two visible-FIR datasets considered, for the task of pedestrian classification, features computed on FIR images performed better than the counterpart computed on visible ones.

**Figure 8:**
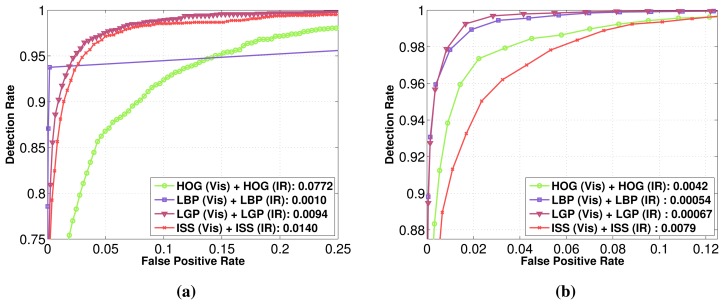
Individual feature fusion between the visible and FIR domains on: (**a**) the RIFIR dataset; (**b**) the ParmaTetravison dataset. The reference point is false positive rate obtained for a classification rate of 90%.

By fusing both spectra, as seen in Figure 8a for RIFIR and Figure 8b for Parma-Tetravision, the false positive rate for a detection rate of 90% is further reduced.

Classification on HOG features computed on the visible and FIR improves by a factor of two for the results, in comparison with just computing those features on the FIR domain, for the RIFIR dataset, and by a factor of five for Parma-Tetravision. For the RIFIR dataset, the same factor of approximately two is obtained for LBP, LGP and ISS features, while on Parma-Tetravision, the factor will be usually equal to or larger than five.

### 5.6. Multi-Modality Multi-Domain Feature Performance Comparison

**Figure 9:**
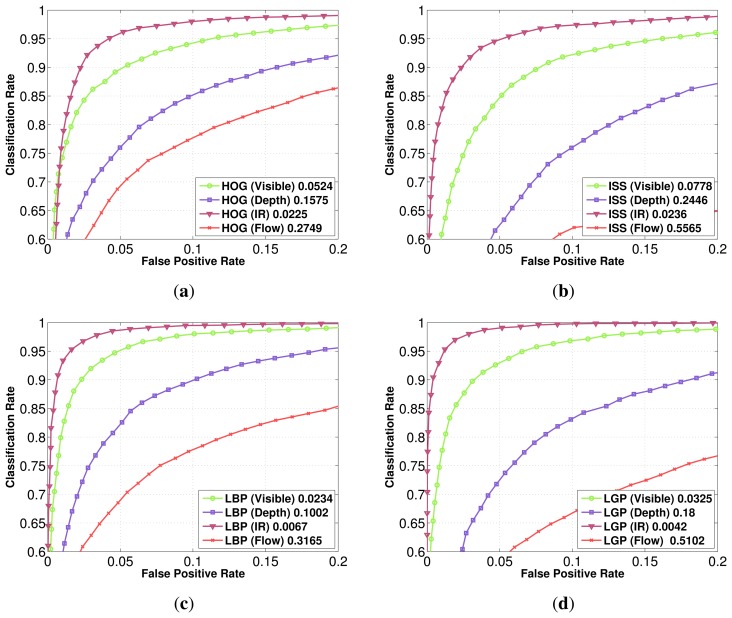
Individual classification (visible, depth, flow and IR) performance of: (**a**) HOG; (**b**) ISS; (**c**) LBP; (**d**) LGP. The reference point is the false positive rate obtained for a classification rate of 90%.

In Figure 9 are presented the performances of each feature family on each modality (intensity, depth, flow) in the visible and FIR domains on the Parma Tetravision dataset. For each feature family, the best performing domain is that of infrared, followed by the visible domain, then by the intensity and depth modalities. The motion features are less accurate because optical flow provides less robust information than depth, especially when a person does not actually move.

The best performing feature family for the visible domain, that of LBP with a factor of two less false positives than HOG features, is quite different than the results obtained on the Daimler Multi-Cue dataset, where HOG features had the best performance.

For the infrared domain, the best performing feature family is LGP, followed closely by LBP. The HOG and ISS features have, for the infrared domain, quite similar performance, but those features have a larger error rate, in comparison with the LGP and LBP ones. Thus, classification with LGP features has a factor of five less false positives than with HOG features.

For the depth modality, the best performing feature family is LBP, followed this time by HOG features. Even if for Daimler Multi-Cue dataset, the ISS features have had the best results for depth, for the Parma-Tetravision, those features are not very robust, having a factor of two more false positives than the LBP features.

As concerns the motion modality, in comparison with the experiments performed on the Daimler Multi-Cue dataset where LGP features gave the best results, on these images, the best performing feature family was HOG. We believe that this variation could be due to the difference in quality of the dense optical flow images among these datasets.

In conclusion, the relative performances of features in a multi-modality multi-domain context vary depending on the quality of sensors, the environment and image processing. Therefore, we decided to fuse all of them, with the exception of features for the flow modality, which, in our case, were not discriminative enough, in order to take advantage of their complementarity.

### 5.7. Multi-Modality Multi-Domain Feature Fusion

**Figure 10:**
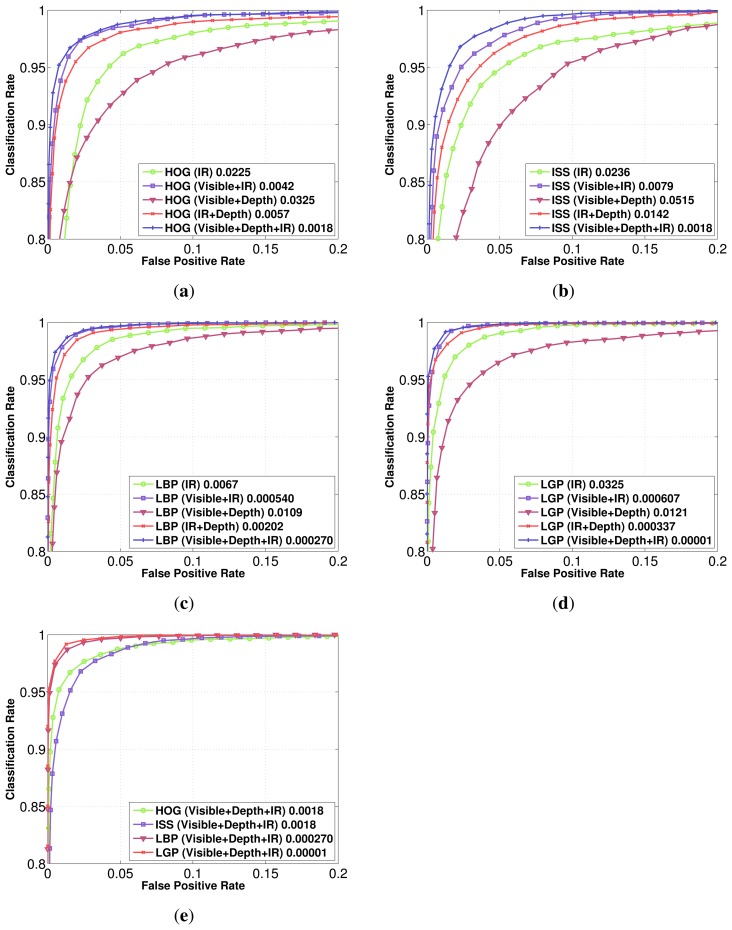
Classification performance comparison for each feature using different modality fusions (visible + IR; visible + depth; IR + depth; intensity + depth + IR) and the best single modality for each feature: (**a**) HOG; (**b**) ISS; (**c**) LBP; (**d**) LGP. In order to highlight differences between different features, in (**e**) is plotted, for comparison, all modality fusions for different features.

We compare for each feature family different fusion schemes: intensity in the visible domain with the infrared one, intensity in the visible domain with the depth modality, the infrared domain with the depth modality, along with the multi-modal multi-domain scheme in the visible, depth and infrared domains. The results obtained on the Parma-Tetravision dataset are given in Figure 10. The fusion of visible and depth lowers the false positive rate for all feature families in comparison with the performance obtained on the visible domain. These results are consistent with those obtained on the Daimler Multi-Cue dataset. It is to be noted that the performances obtained with the visible and depth fusion scheme are still less than those obtained in the infrared domain.

Fusing the infrared domain with the depth modality lowers the false positive rate in comparison with that obtained for the infrared domain. The relative improvement of performance varies from one feature family to another, being approximately 4 for the HOG features, 1.6 for ISS features, 3.3 for LBP and with a staggering factor of 96 for LGP features.

The fusion of the visible and infrared domains with the depth modality provides the overall best results for all feature families on the Parma-Tetravision dataset. This fusion scheme allows similar improvement for the HOG and ISS features. However, the family of local binary features is much more robust, since the fusion of LBP features computed for visible, depth and IR has a factor of nine less false positives than with a classifier trained on the HOG features computed over the same three modalities. The multi-modal multi-domain LGP feature fusion has a factor of over 100 less false positives than with the fused HOG features.

## 6. Discussion

**Figure 11:**
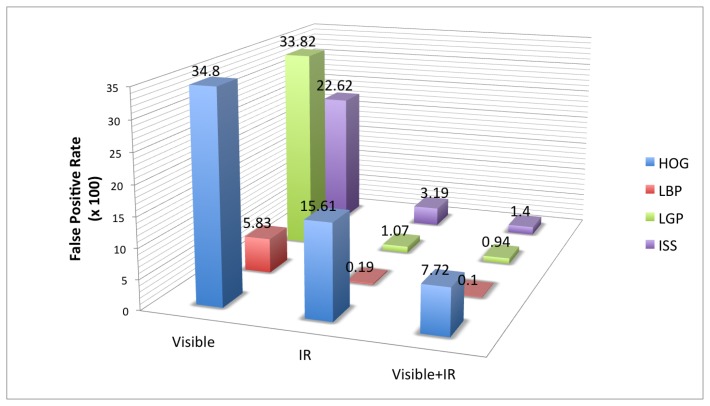
Results overview of the RIFIR dataset.

**Figure 12:**
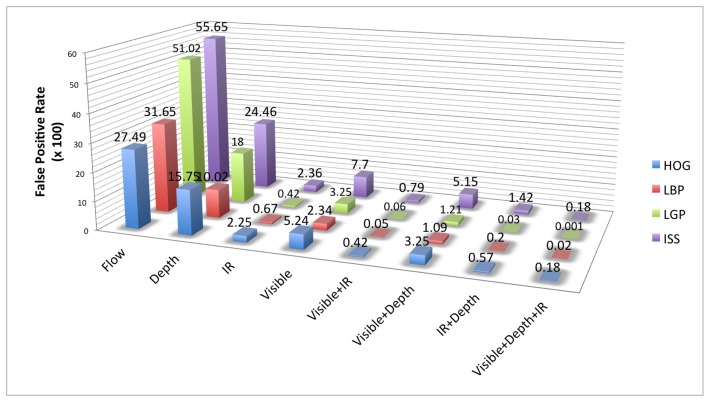
Results overview for the Parma-Tetravision Dataset.

In Figure 11 is presented an overview of the results obtained on the RIFIR dataset, while in Figure 12 is presented an overview of the results obtained on Parma-Tetravision.

In Section 3, we have defined four main questions for which this article wants to provide answers (Q1–Q4). We can definitely answer Q1, since on both datasets acquired in daytime conditions, the FIR spectrum gives better results than the visible one for all of the considered features. If we compare a multi-domain fusion of features, we can observe that the relative gain is quite similar, whatever the feature family, but it might be different from one dataset to another.

To answer Q2, if the state-of-the-art feature families in the visible domain have the same relative performance when tested in the FIR one, we conclude that this is not always the case. For example, for the RIFIR dataset, for the visible domain, the best performing feature is LBP, followed by ISS, but at a great distance. On the same dataset, for FIR domain, the best performing feature is still LBP, followed this time by LGP.

As concerns question Q3, if the ISS feature family is appropriate for pedestrian classification in the considered modalities (intensity, depth, flow) in the visible *versus* FIR domains, we can observe that the ISS features are less robust than LBP or LGP. Despite the good results obtained in our previous work [28] and on Daimler Multi-Cue for depth modality, overall, the LBP/LGP feature families have better performance.

To answer Q4 (what is the relative gain of a multi-domain/multi-modality fusion), we can observe from Figure 12 that no matter what family of feature is employed, a fusion of information from visible, FIR and depth will have an important impact one the false positive rate and will provide the best results from all of the fusion strategies tested.

## 7. Conclusions

This paper has studied the problem of pedestrian classification over two domains, visible and far-infrared, and three modalities, intensity, depth and flow. Moreover, we have proposed a new dataset, RIFIR, which is publicly available, in order to benchmark different features for pedestrian classification. This dataset contains both visible and FIR images, along with correlated pedestrian and non-pedestrian bounding boxes in the two spectra.

A comparison between features computed for the visible and FIR spectrum was performed. For the two tested datasets that contain both visible and FIR images, RIFIR and Parma-Tetravision, the FIR domain provided more discriminative features. Furthermore, the fusion of the two domains will further decrease the false positive error rate.

Various features have different performances across modalities. As a single modality, for the visible domain, intensity has the best performance for all of the features, followed by depth. Even if the fusion of intensity and depth lowers the false positive rate for all features, the intensity values from the FIR domain had consistently lower error rates.

Features computed from the FIR and visible domains are highly complementary, and the use of the two spectra will always lower the error rate. Unfortunately, the information fusion is not straightforward, because two different cameras are used, one for FIR and one for the visible domain; therefore, there will always be a difference in point of view. A correlation method between the two domains is necessary. A possible hardware solution is to construct a camera capable of simultaneously capturing information from both light spectra. Furthermore, as future work, we plan to extend the analysis by benchmarking different pedestrian detection algorithms.

## Figures and Tables

**Table 1:** Dataset comparison for pedestrian classification and detection in FIR images by acquisition setup (surveillance/mobile), infrared spectrum used (FIR/NIR), if they contain also images taken in the visible spectrum, the availability of occlusion labels (Occ.Lab), stereo information (stereo), image resolution, the number of images in the dataset (No.Img.), the number of unique pedestrians annotated (No.U.Ped) and the number of pedestrian bounding boxes (No.Ped.BB).

**Dataset**	**Properties**								

	**Acquisition setup**	**Infrared**	**Visible**	**Occ.Lab**	**Stereo**	**Resolution**	**No.Img.**	**No.U.Ped**	**No.Ped.BB**
ETHZ Thermal Infrared Dataset [32]	Surveillance	FIR	No	No	No	324 × 256	4318	22	6500
OSU Thermal Pedestrian Database [33]	Surveillance	FIR	No	No	No	360 × 240	284	-	984
OSU Color-Thermal Database [34]	Surveillance	FIR	Yes	No	No	320 × 240	17,089	48	-
RGB-NIR Scene Dataset [35]	Surveillance	NIR	Yes	-	No	1024 × 768	477	-	-
OlmedaFIR-Classif [36]	Mobile	FIR	No	No	No	164 × 129	81,529	-	∼16,000
OlmedaFIR-Detection [36]	Mobile	FIR	No	No	No	164 × 129	15,224	-	8400
Parma-Tetravision ^1^ [37]	Mobile	FIR	Yes	Yes ^2^	Yes	320 × 240	18,578	280	∼18,000
**RIFIR (Proposed Dataset)**	Mobile	FIR	Yes	Yes ^2^	No	650 × 480	∼24,000	171	∼20,000

1 Dataset statistics based on our annotations;

2 only two-class occlusion labels available: occluded or not occluded.

**Table 2:** Parma-Tetravision dataset statistics.

	**Sequence Train**	**Sequence Test**	**Overall**
Number of frames	10,240	8338	18,578
Number of unique pedestrians	120	160	280
Number of annotated pedestrian BBs (visible)	11,554	11,451	23,005
Number of annotated pedestrian BBs (IR)	9386	8801	18,187
Number of pedestrian BBs visible in both cameras with height > 32 *px*, and not presented major occlusions	6264	5743	12,007
Number of negative BBs annotated	26,316	14,823	41,139

**Table 3:** RIFIR dataset statistics.

	**Sequence Train**	**Sequence Test**	**Overall**
Number of frames	15,023	9373	24,396
Number of unique pedestrians	138	33	171
Number of annotated pedestrian BBs (Visible)	19,190	7133	26,323
Number of annotated pedestrian BBs (IR)	14,356	6268	20,624
Number of pedestrian BBs visible in both cameras with height > 32 *px*	9202	2034	11,236
Number of negative BBs annotated	25,608	24,444	50,052
